# Supplementary material for: Chemometric Evaluation of Official and Advanced Methods for Detecting Olive Oil Authenticity in Canned Tuna
Source: Foods. 2025 Jul 29;14(15):2667. doi: 10.3390/foods14152667 (PMC12345719; doi:10.3390/foods14152667)
Supplement: Supplementary file 1 [file foods-14-02667-s001.zip › foods-3763070-supplementary.pdf]

Supplementary Material

Chemometric Evaluation of Official and Advanced Methods for Detecting Olive Oil Authenticity in Canned Tuna

Marjeta Mencin[1], Milena Bučar Miklavčič[2], Maja Podgornik[2] and Nives Ogrinc[1*]

[1] *Dept. Environmental Sciences, Jožef Stefan Institute, Jamova 39, 1000 Ljubljana, Slovenia; marjeta.mencin@ijs.si (M.M.); nives.ogrinc@ijs.si (N.O.)*

[2]*Science and Research Centre Koper, Garibaldijeva 1, 6000 Koper, Slovenia; milena.bucarmiklavcic@zrs-kp.si (M.B.M); maja.podgornik@zrs-kp.si (M.P.)*

*Correspondence: nives.ogrinc@ijs.si; Tel.: 00386 1 5885387

Table S1. Mean values for fatty acids in olive oil from canned tuna samples (OL-01 – OL-10) present in lower contents.

	Fatty acids (%, w/w)							
**Sample**	**myristic**	**heptadecanoic**	**heptadecenoic**	**linolenic**	**arachidic**	**gadoleic**	**behenic**	**lignoceric**
Limits*	≤ 0.03	≤ 0.4	≤ 0.6	≤ 1.0	≤ 0.6	≤ 0.5	≤ 0.2	≤ 0.2
OL-01	0.04	0.06	0.1	0.67	0.4	0.28	0.12	0.07
OL-02	0.03	0.05	0.09	0.65	0.39	0.27	0.1	0.06
OL-03	0.04	0.07	0.1	0.6	0.38	0.28	0.1	0.08
OL-04	0.04	0.07	0.11	0.72	0.4	0.3	0.11	0.07
OL-05	0.02	0.07	0.11	0.65	0.41	0.28	0.14	0.08
OL-06	0.03	0.05	0.09	0.72	0.38	0.24	0.11	0.07
OL-07	0.04	0.08	0.1	0.61	0.38	0.28	0.09	0.04
OL-08	0.02	0.07	0.1	0.69	0.39	0.29	0.11	0.06
OL-09	0.04	0.07	0.09	0.65	0.4	0.26	0.14	0.02
OL-10	0.02	0.08	0.11	0.61	0.38	0.29	0.12	0.06

* Limits established by the current EU Regulation 2022/2104 for olive oil.

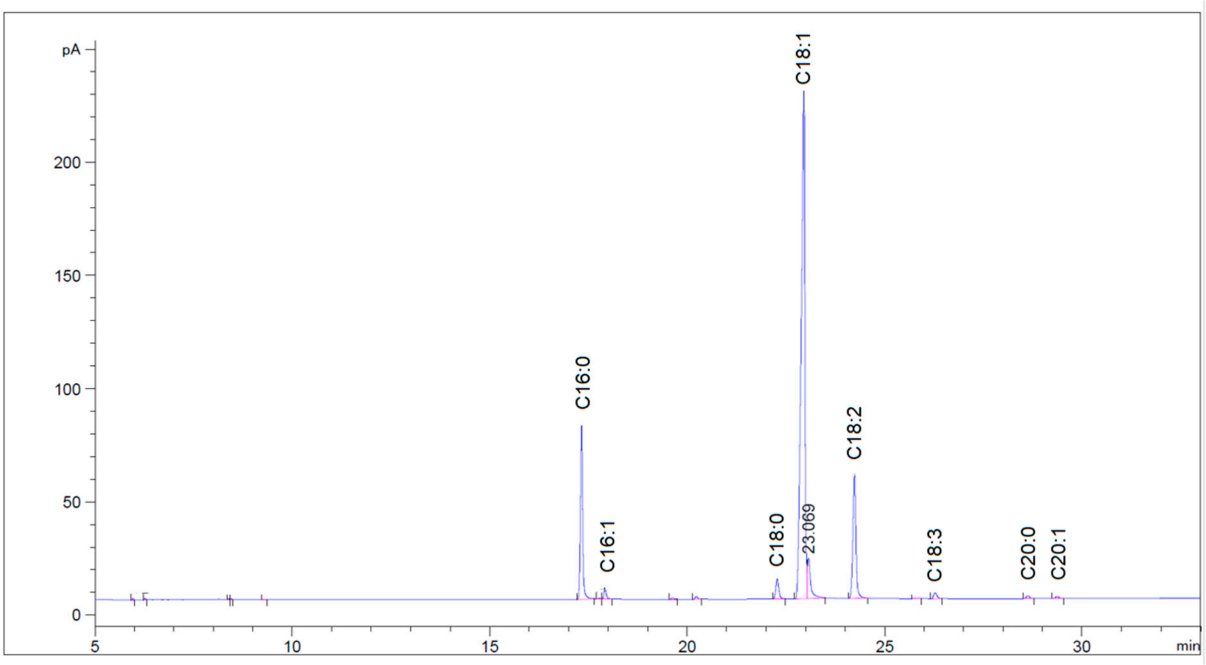

Figure S1. GC-FID chromatogram of a representative olive oil sample from canned tuna showing the separation of the fatty acid methyl esters (palmitic acid (C16:0): 17.3 min; palmitoleic acid (16:1): 17.9 min; stearic acid (C18:0): 22.3 min; oleic acid (C18:1): 22.9 min; linoleic acid (C18:2): 24.2 min; linolenic acid (C18:3): 26.3 min; arachidic acid (C20:0): 28.6 min; gadoleic acid (20:1): 29.4 min).

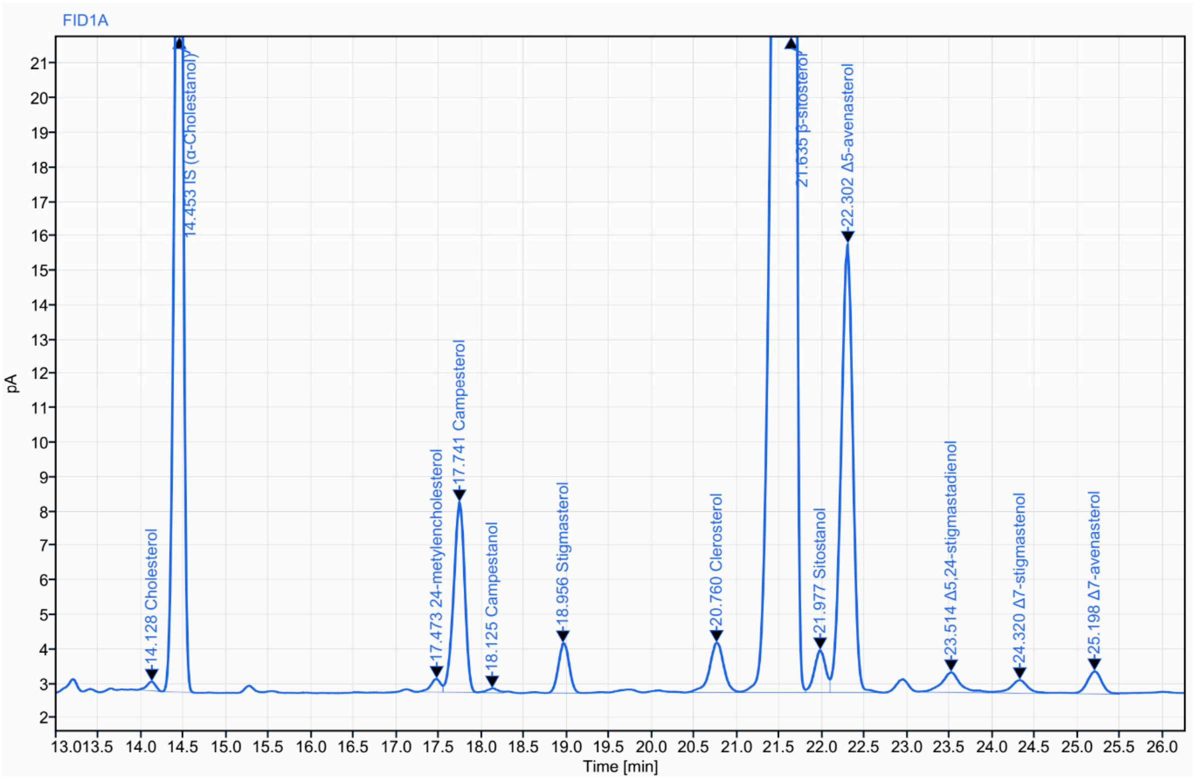

Figure S2. GC-FID chromatogram of a representative olive oil sample from canned tuna showing the separation of the sterols (Cholesterol: 14.13 min; 24-methylene-cholesterol: 17.47 min; Campesterol: 17.74 min; Campestanol: 18.13 min; Stigmasterol: 18.96 min; Clerosterol: 20.76 min; β-sitosterol: 21.64 min; Sitostanol: 21.98 min; Δ-5-avenasterol: 22.30 min; Δ-5,24-stigmastadienol: 23.51 min; Δ-7-stigmastenol: 24.32 min; Δ-7-avenasterol: 25.20 min).

A

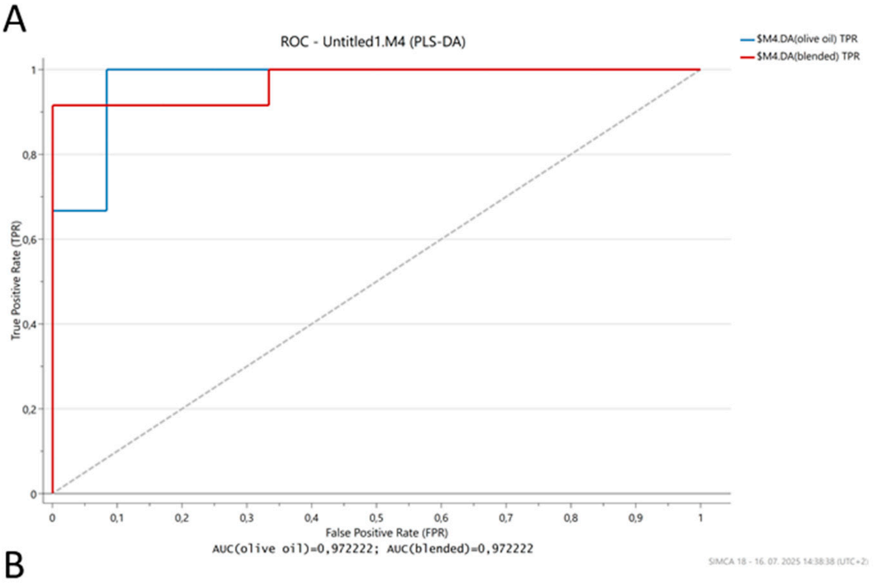

B

Classes	Members	Correct	olive oil	blended	No class (YPred <= 0)
**olive oil**	6	66,67%	4	2	0
**blended**	12	100%	0	12	0
**No class**	0		0	0	0
Total	18	88,89%	4	14	0
Fisher's prob.	0,0049				

Figure S3: A) Receiver operating characteristic (ROC) curve for classification of olive oil samples (authentic and blended) using PLS-DA, based on fatty acid profiles. The Area Under the Curve (AUC) values are 0.972 for both authentic (olive oil) and adulterated (blended) samples. B) Misclassification table showing the percentage of correctly classified samples, confirming good accuracy of the PLS-DA model.

A

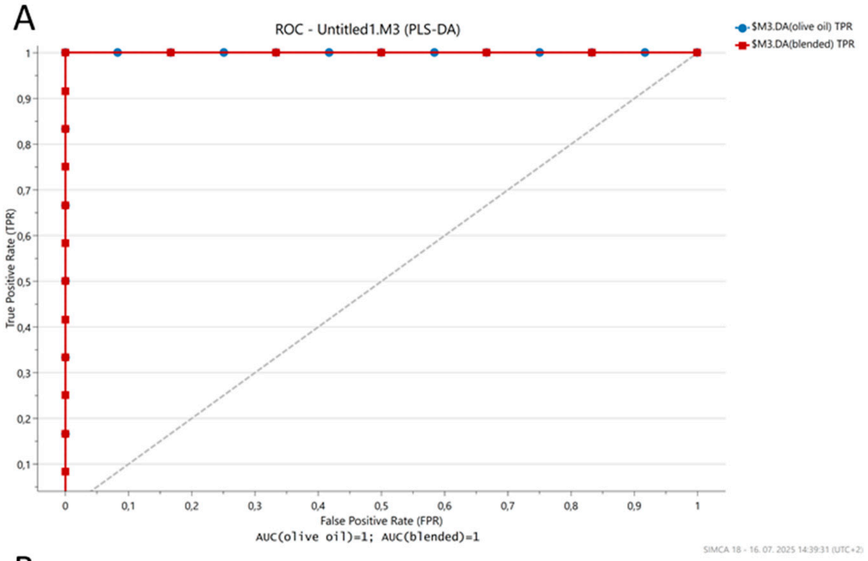

B

Classes	Members	Correct	olive oil	blended	No class (YPred <= 0)
**olive oil**	6	100%	6	0	0
**blended**	12	100%	0	12	0
**No class**	0		0	0	0
Total	18	100%	6	12	0
Fisher's prob.	5,4e-05				

Figure S4: A) Receiver operating characteristic (ROC) curve for classification of olive oil samples (authentic and blended) using PLS-DA, based on carbon stable isotope analysis of fatty acids. The Area Under the Curve (AUC) values are 1.0 for both authentic (olive oil) and adulterated (blended) samples. B) Misclassification table showing the percentage of correctly classified samples, confirming high accuracy of the PLS-DA model.

A

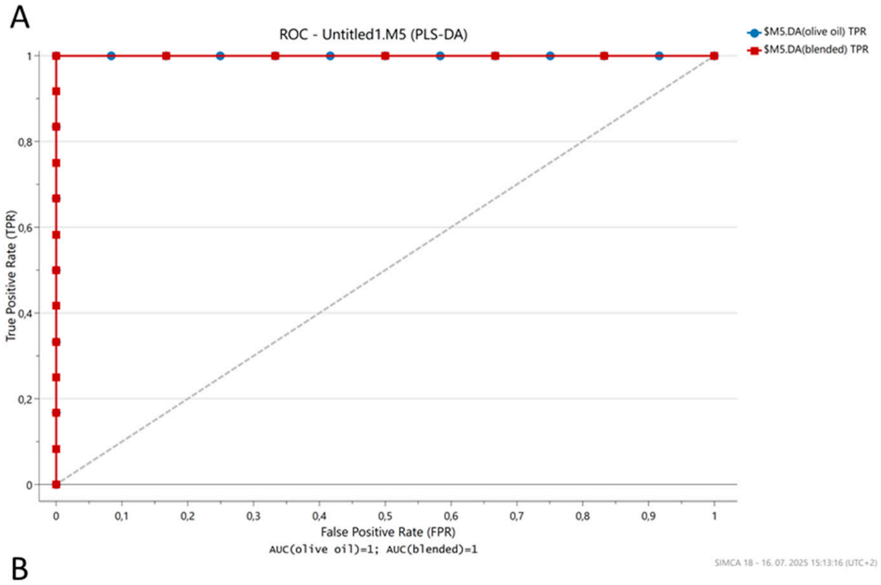

B

Classes	Members	Correct	olive oil	blended	No class (YPred <= 0)
**olive oil**	6	100%	6	0	0
**blended**	12	100%	0	12	0
**No class**	0		0	0	0
Total	18	100%	6	12	0
Fisher's prob.	5,4e-05				

Figure S5: A) Receiver operating characteristic (ROC) curve for classification of olive oil samples (authentic and blended) using PLS-DA, based on fatty acid profiles and carbon stable isotope analysis of fatty acids. The Area Under the Curve (AUC) values are 1.0 for both authentic (olive oil) and adulterated (blended) samples. B) Misclassification table showing the percentage of correctly classified samples, confirming high accuracy of the PLS-DA model.
